# miR-105/93-3p promotes chemoresistance and circulating miR-105/93-3p acts as a diagnostic biomarker for triple negative breast cancer

**DOI:** 10.1186/s13058-017-0918-2

**Published:** 2017-12-19

**Authors:** Hao-Yi Li, Jui-Lin Liang, Yao-Lung Kuo, Hao-Hsien Lee, Marcus J. Calkins, Hong-Tai Chang, Forn-Chia Lin, Yu-Chia Chen, Tai-I Hsu, Michael Hsiao, Luo-Ping Ger, Pei-Jung Lu

**Affiliations:** 1 Institute of Clinical Medicine, College of Medicine, National Cheng Kung University, Tainan, Taiwan; 2 Department of General Surgery, Chi-Mei Medical Center, Liouying, Tainan, Taiwan; 3 Department of General Surgery, National Cheng Kung University Hospital, Tainan, Taiwan; 4 Department of Surgery, College of Medicine, National Cheng Kung University, Tainan, Taiwan; 5 Department of Surgery, Kaohsiung Veterans General Hospital, Kaohsiung, Taiwan; 6 Department of Radiation Oncology, National Cheng Kung University Hospital, Tainan, Taiwan; 7 Division of General Surgery, Department of Surgery, Kaohsiung Veterans General Hospital, Kaohsiung, Taiwan; 8 Department of Orthopedics, National Cheng Kung University Hospital, Tainan, Taiwan; 9 Department of Orthopedics, E-DA Hospital, Kaohsiung, Taiwan; 10 Genomics Research Center, Academia Sinica, Taipei, Taiwan; 11 Department of Biochemistry, College of Medicine, Kaohsiung Medical University, Kaohsiung, Taiwan; 12 Department of Medical Education and Research, Kaohsiung Veterans General Hospital, Kaohsiung, Taiwan; 13 Department of Clinical Medicine Research, National Cheng Kung University Hospital, Tainan, Taiwan; 14 Graduate Institute of Medicine, Kaohsiung Medical University, Kaohsiung, Taiwan

**Keywords:** miR-105, miR-93-3p, Biomarker, Cisplatin, Drug resistance, Triple negative breast cancer

## Abstract

**Background:**

Triple negative breast cancer (TNBC) lacks both early detection biomarkers and viable targeted therapeutics. Moreover, chemotherapy only produces 20–30% pathologic complete response. Because miRNAs are frequently dysregulated in breast cancer and have broad tissue effects, individual or combinations of circulating miRNAs may serve as ideal diagnostic, predictive or prognostic biomarkers, as well as therapeutic targets. Understanding the role and mechanism of dysregulated miRNAs in TNBC may help to develop novel diagnostic and prognostic strategy for TNBC patients.

**Methods:**

The miRNA array profiles of 1299 breast cancer patients were collected from the Metabric database and subjected to analysis of the altered miRNAs between TNBC and non-TNBC. In Student’s *t*-test and Kaplan-Meier analysis, four upregulated miRNAs correlated with poor survival in TNBC but not in non-TNBC. Four miRNAs were manipulated in multiple cell lines to investigate their functional role in carcinogenesis. From these results, we studied miR-105 and miR-93-3p in greater detail. The level of miR-105 and miR-93-3p were evaluated in 25 breast cancer tumor tissues. In addition, the diagnostic utility of circulating miR-105 and miR-93-3p were examined in 12 normal and 118 breast cancer plasma samples by ROC curve construction.

**Results:**

miR-105 and miR-93-3p were upregulated and correlated with poor survival in TNBC patients. Both miR-105 and miR-93-3p were found to activate Wnt/β-catenin signaling by downregulation of SFPR1. By this action, stemness, chemoresistance, and metastasis were promoted. Importantly, the combination of circulating miR-105/93-3p may serve as a powerful biomarker for TNBC, even in early-stage disease.

**Conclusions:**

miR-105/93-3p activates Wnt/β-catenin signaling by downregulating SFRP1 and thereby promotes stemness, chemoresistance, and metastasis in TNBC cells. Most importantly, combined circulating miR-105/93-3p levels represent a prime candidate for development into a diagnostic biomarker for both early- and late-stage TNBC.

**Electronic supplementary material:**

The online version of this article (doi:10.1186/s13058-017-0918-2) contains supplementary material, which is available to authorized users.

## Background

Triple negative breast cancer (TNBC) accounts for 15–20% of all breast cancers and is defined by a lack of estrogen receptor (ER), progesterone receptor (PR), and human epidermal growth factor receptor 2 (HER2) [1]. Pathologically, TNBC is usually characterized by a ductal or mixed histology, high grade, and high proliferation. Patients with TNBC have a poor prognosis, shorter overall and disease-free survival and higher risk of distal recurrence compared to those with other types of breast cancer [2, 3]. Since there is a lack of effective hormone or targeted therapies for TNBC, surgery and chemotherapy are the standard treatment [4]. Although neoadjuvant chemotherapy can result in a 20–30% pathologic complete response (pCR), TNBC patients have lower 3-year overall survival rates compared with non-TNBC breast cancer patients [5]. In fact, among TNBC patients receiving neoadjuvant chemotherapy, only those with pCR show improved survival. The remaining 70–80% of TNBC patients have residual disease after neoadjuvant chemotherapy and suffer from the high risk of relapse and poor survival, especially in the first three years [5, 6]. Therefore, developing methods to improve the pCR rate in TNBC is currently a high priority, which will most likely require the identification of novel therapeutic targets. In addition, TNBC is often diagnosed late, with a high histological grade. This late diagnosis is highly problematic because the 5-year survival rate is dramatically decreased from stage II (76%) to stage III (45%) cancers [7]. However, there are currently no early diagnostic biomarkers for TNBC. Thus, along with novel target discovery, identification, and development of early detection biomarkers for TNBC is another critical undertaking.

MicroRNAs (miRNAs) are short non-coding RNAs that negatively regulate targeted mRNAs by destabilization or translational repression. Accumulating evidence indicates that dysregulated miRNAs may modulate various signaling pathways, including Wnt/β-catenin, to promote breast cancer initiation, progression, metastasis and chemoresistance [8–12]. Recently, miRNAs have also been reported to be useful subtype-specific diagnostic, predictive or prognostic biomarkers in breast cancer. Interestingly, miRNAs can be secreted by solid tumors into circulation and afterward, may be detected in bodily fluids such as blood, plasma, and serum [13]. These circulating miRNAs can be incorporated into exosomes or interact with proteins like Agonature2 (Ago2) to increase stability in blood [14, 15]. A number of studies have demonstrated that circulating miRNAs may be useful as either diagnostic, predictive, or prognostic biomarker in various cancers including breast cancer [16–18]. Circulating miRNAs are ideal for this purpose because of their stability, ease of measurement, and because collection does not require an invasive biopsy.

The purpose of this study was to identify miRNAs that are specifically associated with poor survival in TNBC and determine the underlying mechanism by which they contribute to carcinogenesis. We found that miR-301b, miR-181a-3p, miR-105, and miR-93-3p expression is specifically associated with poor survival in TNBC. Importantly, circulating miR-105 and miR-93-3p were significantly and specifically upregulated in TNBC patients and have strong predictive power for the TNBC subtype, even in early stage cancers. Moreover, overexpression of miR-105 and miR-93-3p can increase stemness, chemoresistance, and metastasis in TNBC cells by activating Wnt/β-catenin signaling through downregulation of secreted frizzled related protein 1 (SFRP1). Overall, we uncovered a novel mechanism of miR105/93-3p-mediated chemoresistance and established that circulating miR-105/93-3p can serve as a powerful diagnostic, predictive, and prognostic biomarker for TNBC.

## Methods

### miRNA expression profiling and statistical analysis

A total of 1299 miRNA microarrays and mRNA expression profiles from breast cancer patients using fresh frozen tissue were obtained from the Metabric database [19]. The pool was subdivided into TNBC (ER-/PR-/HER2-) and non-TNBC groups (any of the ER, PR or HER2 receptors that showed positive signal). Candidate miRNAs were selected based on differential miRNA expression levels between TNBC and non-TNBC, first compared by Student’s *t* test and followed by Kaplan-Meier analysis. The median expression level from all 1299 samples was used as a cutoff point for separating high or low miRNA expression. All tests were two-sided and *p* value of less than 0.05 was considered to be statistically significant.

### Cell culture

All of the cells were purchased from American Type Culture Collection (Manassas, VA, USA) at the end of 2012. All cell lines used in this manuscript were stored in Cryosystem until March 2015. The cells were first thawed according to the standard protocol and subsequently subcultured by the following protocol to maintain the cells till experimental usage. The MCF 10A cell line was a non-tumorigenic epithelial cell line; MB-361, MCF-7, BT-483, AU565, and SkBR3 were non-TNBC cell lines; MB-231 (MDA-MB-231), Hs578T, HCC1599, HCC1806, HCC1937, BT-549, DU4475, and HCC70 were belonged to TNBC cell lines. MB-231 (MDA-MB-231) and 293 T cells were maintained in DMEM, 10% Cosmic Calf Serum (CCS; HyClone, Logan, UT, USA) and 1% penicillin/streptomycin (P/S) solution (Caisson Labs, Smithfield, UT, USA). HCC70 and HCC1937 cells were maintained in RPMI, 10% CCS and 1% P/S solution; The BT-549 cells were cultured with RPMI, 10% CCS, 1% P/S solution and 8 ug/ml insulin. All the experiments in the cell model were completed in November 2016. The cells were provided by Michael Hong-Shen Hsiao (Michael Hsiao), who is our co-author on this manuscript.

### Clinical specimens

Human breast specimens were obtained from Kaohsiung Veterans General Hospital (Kaohsiung, Taiwan), Chi Mei Medical Center (Tainan, Taiwan) and National Cheng Kung University Hospital (Tainan, Taiwan). All patients gave informed consent and the protocol was approved by the institutional review boards of all three hospitals. The tissue samples were collected from the tissue bank. From each patient, a plasma sample was collected during their planned treatment. The 25 tissue (NT pair) and 130 plasma samples were stored in liquid nitrogen until use. The patients were subdivided into TNBC (ER-/PR-/HER2-) and non-TNBC groups (any of the ER, PR or HER2 receptors that showed positive signal).

### Stem-loop RT-qPCR for detection of miRNA

Primers targeting the stem-loop region of miRNA were designed for reverse transcription of miRNA to cDNA, as previously described [20]. For qPCR, a specific forward primer was designed for each miRNA and reverse primers were targeted to the stem-loop sequence. Expression levels were analyzed by qPCR using SYBR Green master mix (KAPA Biosystems, Wilmington, MA, USA) and the non-coding small nuclear RNA, U6 was used as an internal control.

### Cisplatin treatment and MTT assay

The mimic miRNAs and miRNA antagomirs of miR-301b, miR-181a-3p, miR-105, miR-93-3p and negative control were purchased from TOOLS company. For overexpression or silence miRNAs in TNBC cells, the four mimic miRNAs or miRNA antagomirs were transfected respectively or combined. The miRNA-overexpressing or silencing TNBC cells were seeded at 1 × 10^4^ cells per well in 96-well plate. After cells attached, the cells were washed twice with HBSS and then transferred to cisplatin-containing culture medium. After cisplatin (Sigma-Aldrich, St, Louis, MO, USA, 479306) treatment for 72 hours, MTT (MDBio, Inc, Gaithersburg, MD, USA) was diluted in culture medium to final 20% v/v, and 100 μl of the solution was added to each well for 30 minutes. DMSO was used to dissolve crystals and OD570 nm was measured.

### Bioinformatic prediction of miRNA target genes and related cell signaling pathways

The RNA22, RNAhybrid and Targetscan online tools were utilized to predict the target genes for miR-105 and miR-93-3p. The DAVID website was then used to analyze which cell signaling pathways are likely to be modulated by the target genes of miR-105/93-3p. In addition, 100 mRNAs that were most closely associated with miR-105 or miR-93-3p by Pearson’s correlation analysis were evaluated by Ingenuity Pathway Analysis (IPA).

### Immunoblotting

Protein lysates were loaded onto SDS-polyacrylamide gels for electrophoresis and transferred to a PVDF membrane (EMD Millipore, Billerica, MA, USA) and incubated with indicated primary antibodies, including GFP (Santa Cruz Technology, Dallas, TX, USA sc-9996), Flag (Sigma-Aldrich, M2), SFRP1 (Cell Signaling Technology, Danvers, MA, USA, #3534), Tubulin (Santa Cruz Technology, sc-8035) and GAPDH (GeneTex, GTX100118) for 2 hours at room temperature. The membrane was washed with TBST three times for 10 minutes, incubated with the secondary antibodies and HRP-conjugated signaling detected by X-ray films.

### Ago2-mediated RNA-immunoprecipitation

A Flag-Ago2 expression construct was combined with mimic miRNAs of miR-105 or miR93-3p (TOOLS), transfected into 293 T cells and incubated for 48 hours. The cell lysates were harvested using RIPA buffer, containing protease inhibitors and RNase inhibitor. Flag antibody (Santa Cruz Technology, sc-9996) was pre-incubated with Protein G-agarose beads (EMD Millipore, 16-266) overnight at 4 °C and washed three times with IP wash buffer. The cell lysates were incubated with pre-conjugated antibodies overnight at 4 °C and washed three times with IP wash buffer. The pellet from immunoprecipitation was resolved in TRIsure reagent (BIOLINE, Taunton, MA, USA, BIO-38033) for RNA extraction and PCR.

### Circulating miRNA extraction and detection

Circulating miRNAs were extracted using Direct-zol™ RNA MicroPrep Kit (Zymo Research Irvine, CA, USA) and 50 ng of total RNAs were reverse transcribed into cDNA based on poly A-tail directed reverse transcription as previously described [21]. Expression levels were analyzed by qPCR using SYBR Green master mix (KAPA) and U6 was used as the internal control.

### Statistical analyses

All observations were confirmed by at least three independent experiments. Data were expressed as means ± SEM. The associated with overall survival was analyzed using the log-rank Kaplan-Meier analysis. Statistical comparisons of the results were made using Student’s *t* test. All tests were two-sided, and *p* value of less than 0.05 was considered to be statistically significant. The receiver operating characteristic (ROC) curve was used to examine the predictive power, and the area under the ROC curve (AUC) was represented the predictive power. The cutoff point of ROC curve was chosen by Youden’s Index [22]. IBM SPSS version 20.0 (IBM Corp, Armonk, NY, USA) and GraphPad Prism 6 software (GraphPad Software, San Diego, CA, USA) were used to analyze data.

## Results

### Identification and validation of miRNAs associated with poor survival in TNBC

**Fig. 1:**
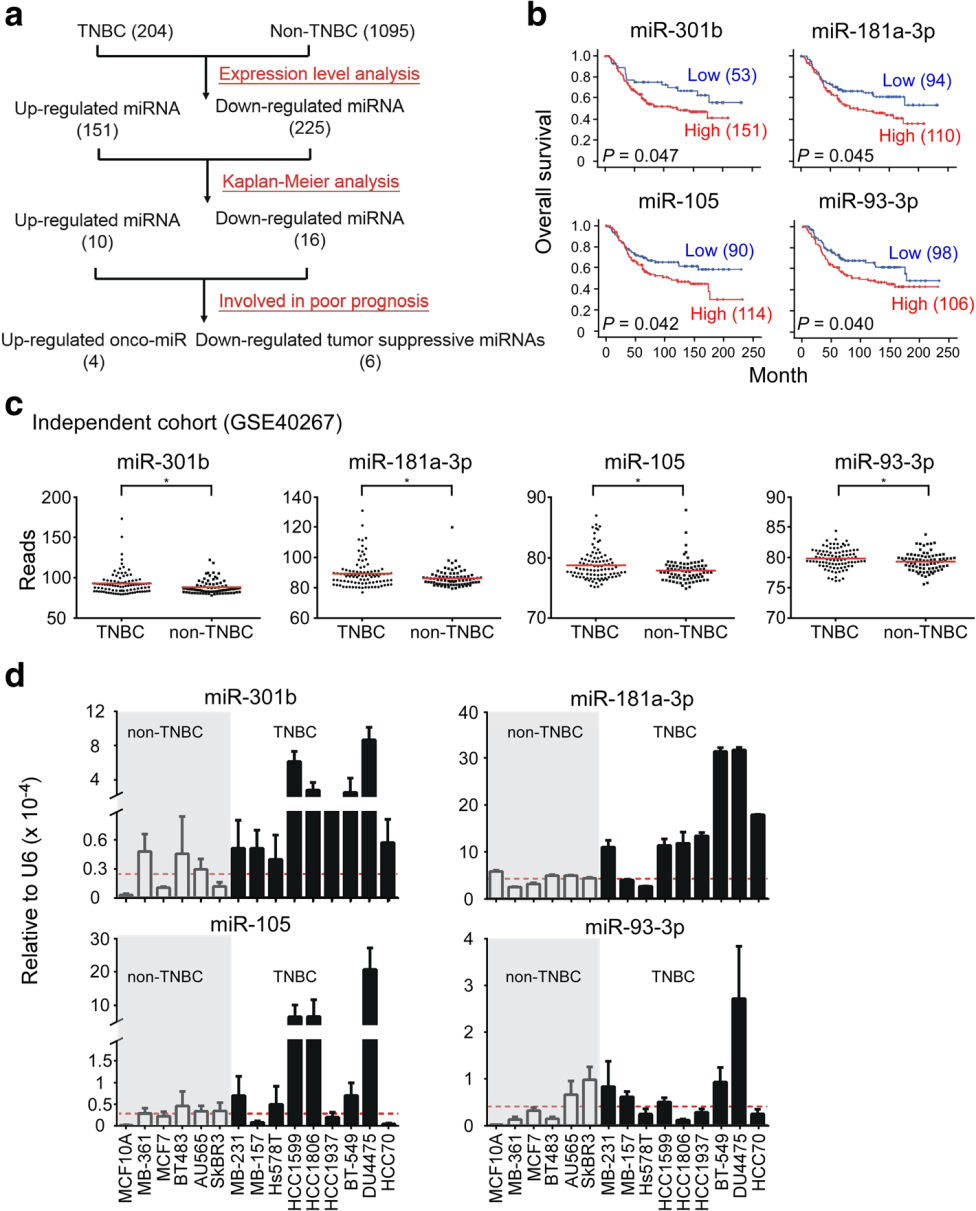
Identification and validation of four oncomiRs in TNBC. **a** Illustration of the strategy to identify miRNAs that are associated with poor survival in TNBC patients. **b** Four microRNAs were found to have elevated expression that was associated with poor overall survival in 204 triple negative breast cancer patients by Kaplan-Meier analysis. **c** Expression levels of the four upregulated oncomiRs were examined in an independent cohort (GSE40267, *N* = 173). **d** Expression levels of the four oncomiRs were examined in 15 breast cancer cell lines, including MCF10A, five non-TNBC and nine TNBC cell lines by qPCR. The relative miRNA expression levels were normalized to U6. Mean ± SEM; ^*^ *P* < 0.05

In order to identify miRNAs that were associated with poor survival in TNBC, miRNA microarray profiles of 1299 patients were collected from the Metabric database [19, 23] and analyzed to examine differential expression between TNBC and non-TNBC breast cancer patients. To validate the reliability of the cohort, several clinical and pathological characteristics of TNBC and non-TNBC groups were quantified (Table 1). The TNBC group had shorter survival, larger tumor size, and higher grade compared with non-TNBC (Table 1), consistent with previous studies [2]. Next, miRNA expression levels were compared between 204 TNBC and 1095 non-TNBC by Student’s *t*-test followed by Kaplan-Meier analysis (Fig. 1a). From the Student’s *t*-test, 376 of 616 miRNAs levels were found to be significantly different between groups, including 151 upregulated and 225 downregulated miRNAs in the TNBC group (Additional file 1: Figure S1A). However, only ten of these 376 miRNAs were significantly (*P* < 0.05) associated with poor survival in TNBC patients, according to Kaplan-Meier analysis (Additional file 2: Table S1). Among them, four were upregulated oncomiRs (miR-301b, miR-181a-2-3p, miR-105-5p and miR-93-3p) and six were downregulated tumor suppressive miRNAs (miR-7-1-3p, miR-135a, miR-628-5p, miR-638, miR-3173, and miR-4245) were significantly associated with poor survival in TNBC patients but not in non-TNBC patients (Fig. 1b and Additional file 1: Figure S1B–D). To verify the expression level differences of these miRNAs in TNBC patients, an independent cohort obtained from a GEO dataset (GSE40267) containing 173 patients (94 TNBC and 79 non-TNBC) was analyzed for the expression level of candidate miRNAs [24] (Fig. 1c and Additional file 1: Figure S1E). The results confirmed that all four oncomiRs obtained from the Metabric database were upregulated in TNBC, whereas, the only downregulated miRNA to be confirmed was miR-628-5p. To determine the expression levels of miR-301b, miR-181a-3p, miR-105, and miR-93-3p in 15 breast cancer cell lines, quantitative PCR was used to examine nine TNBC, five non-TNBC, and MCF-10A cell lines. The data showed that miR-301b, miR-181a-3p, miR-105, and miR-93-3p, were indeed highly expressed in TNBC cell lines compared to non-TNBC cell lines and MCF-10A (Fig. 1d). Taken together, our analyses identified four oncomiRs, miR-301b, miR-181a-3p, miR-105, and miR-93-3p that were significantly upregulated and associated with poor survival in TNBC patients.

**Table 1:** Clinical features of TNBC and non-TNBC patients

	Non-TNBC	TNBC	*P*
Survival (months, median)	84.8	65.85	0.006^#^
Tumor size (units, median)	22	25	0.153^#^
Grade			<0.001^*^
1	103	3	
2	475	27	
3	462	165	
Unknown	55	9	
Stage			0.425
0–2	863	155	
3–4	88	22	
Unknown	144	27	
Lymph nodes			0.096
No	606	100	
Yes	489	104	
Distal metastasis			0.578
No	799	145	
Yes	296	59	

*TNBC* triple negative breast cancer

^#^Student’s *t* test

^*^Chi-square test

### miR-105 and miR-93-3p promoted chemoresistance in TNBC

**Fig. 2:**
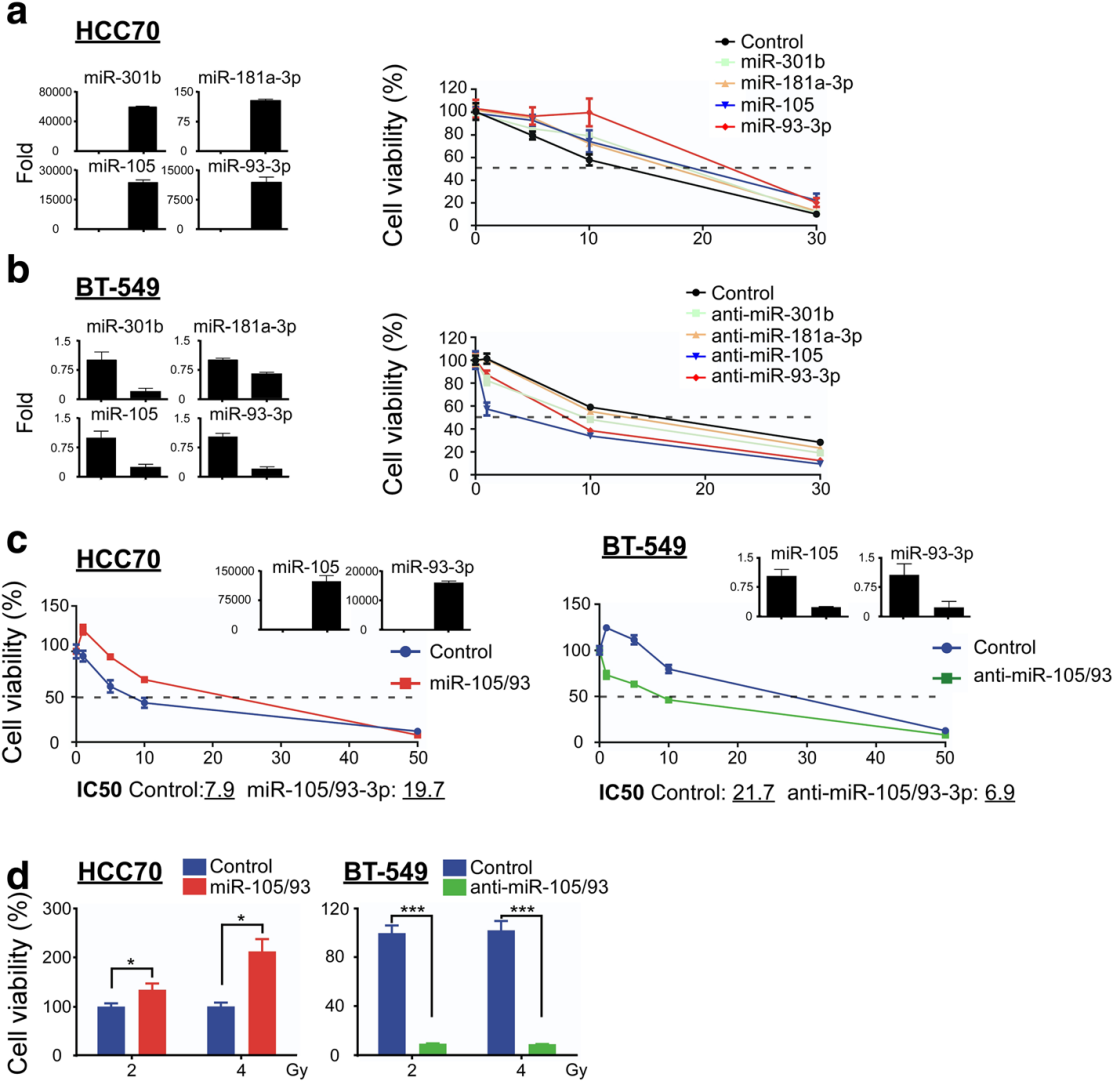
miR-105 and miR-93-3p promote cisplatin/CCRT resistance in TNBC cells. To determine the effect of miRNAs on cisplatin response, four oncomiRs were individually (**a**) overexpressed in HCC70 cells or (**b**) silenced in BT-549 cells and cells were treated with indicated cisplatin doses for 72 hours before cell viability was measured by the MTT assay. **c** Co-overexpression or co-knockdown of miR-105/93-3p in HCC70 and BT-549, respectively, was performed and cells were treated with the indicated cisplatin doses for 72 hours to before viability was measured with the MTT assay. **d** Cells were treated with the indicated doses of radiation combined with 5 μM cisplatin. CCRT response was measured in miR-105/93-3p co-overexpressing-HCC70 cells or co-silenced-BT-549 cells. The cell viabilities were normalized to control. Mean ± SEM; ^*^ *P* < 0.05, ^***^ *P* <0.001

There is substantial evidence that chemoresistance, metastasis, and stemness are major contributors to poor survival in breast cancer patients [2, 5, 25]. To explore the oncogenic roles of the four oncomiRs identified above, individual miRNAs were ectopically expressed in HCC70/MB-231 cells or silenced in BT-549/HCC1937 cells and confirmed by qPCR (shown in Fig. 2a–b, left panel). First, our results showed that neither overexpression nor silencing of any of the four miRNAs affected proliferation ability by foci formation and MTT proliferation assays (Additional file 1: Figure S2A and S2B). Next, we found that ectopic overexpression of miR-105 or miR-93-3p, but not miR-301b and 181a-3p can increase cellular migration and invasion ability by approximately 1.5-fold in a Boyden chamber assay. Conversely, only silencing of miR-93-3p significantly decreased the cellular invasion ability (approximately 80% of control group, *P* < 0.001), suggesting miR-105 and miR-93-3p may functionally promote tumor malignancy in TNBC (Additional file 1: Figure S2C–D).

To investigate whether the four oncomiRs contribute to chemoresistance, individual miRNAs were overexpressed or silenced in four TNBC cell lines. Cisplatin-induced cell death was then examined by MTT assay. Figure 2a and Additional file 1: Figure S3A showed the IC50 value increased from 11.1 μM to 16.6 μM and 24.4 μM in HCC70 cells upon miR-105 and miR-93-3p overexpression, respectively; furthermore, the IC50 increased from 16.5 μM to 39.6 μM (miR-105) and 41.4 μM (miR-93-3p) in MB-231 cells. The IC50 values of miR-301b- and miR-181a-3p-overexpressing cells did not change significantly in either HCC70 or MB-231 cells (miR-301b/181a-3p = 15.1 μM/14.5 μM in HCC70 cells; miR-301b/181a-3p = 24.0 μM/12.3 μM in MB-231 cells). Figure 2b and Additional file 1: Figure S3B showed the IC50 values decreased from 14 μM to 1.9 μM and 6.2 μM when treated with antagomiRs of miR-105 and miR-93-3p, respectively, in BT-594 cells; moreover, the IC50 decreased from 74.4 μM to 66.1 μM (miR-105) and 32.2 μM (miR-93-3p) in HCC-1937 cells. Again, the IC50 values of miR-301b and miR-181a-3p silenced cells were not different than controls in either BT-549 or HCC1937 cells (miR-301b/181a-3p = 7.5 μM and 12.1 μM in BT-549 cells; miR-301b/181a-3p = 84.9 μM and 81.2 μM in HCC1937 cells). These results supported the hypothesis that both miR-105 and miR-93-3p had functional effects on chemoresistance. Furthermore, co-overexpression of miR-105/93-3p (Fig. 2c upper left inserted panel) can increase the IC50 value of cisplatin-induced cell death from 7.9 μM to 19.7 μM in HCC70, whereas, co-knockdown of miR-105/93-3p (Fig. 2c upper right inserted panel) can reduce the IC50 value of cisplatin-induced cell death from 21.7 μM to 6.9 μM in BT-549 cells (Fig. 2d). Similar effects can also be observed in HCC1937 cells, where the IC50 value decreased from 78.9 μM to 16 μM upon miR-105/93-3p co-knockdown (Additional file 1: Figure S3C). In addition, co-overexpression of miR-105/93-3p promoted concurrent chemoradiotherapy (CCRT) resistance under 2Gy (*P* = 0.045) or 4 Gy (*P* = 0.003) radiation treatment, whereas, miR-105/93-3p silencing enhanced CCRT sensitivity under 2 Gy (*P* < 0.001) or 4 Gy (*P* < 0.001) radiation treatment (Fig. 2e). Stemness has been reported to contribute to drug resistance, CCRT resistance, and metastasis [26]. Therefore, a mammosphere formation assay was used to investigate the effect of miR-105 and miR-93-3p on cancer stemness. Knockdown of miR-105/93-3p together significantly reduced sphere size and numbers (from 72.7 ± 1.7 to 40.3 ± 2.7 spheres, *P* < 0.001) in BT-549 cells (Additional file 1: Figure S3D), suggesting that elevated expression of miR-105/93-3p may enhance chemoresistance and metastasis in TNBC patients. In accordance with this hypothesis, high expression of miR-105/93-3p was significantly correlated with distal metastasis in TNBC patients and Cox regression analysis revealed that miR-105/93-3p showed a higher hazard ratio (HR) than miR-301b/181a-3p (multivariate HR: miR-105/93-3p = 1.60/2.16; miR-301b/181a-3p = 1.44/1.02) (Table 2 and Additional file 3: Table S2). Collectively, these results suggest that elevated miR-105/93-3p promotes chemoresistance and may together with increased stemness, and metastasis properties to contribute the poor survival of TNBC patients.

**Table 2:** Clinical features of miR-93-3p/105 and miR-301b/181a-3p TNBC patients

	miR-301b/181a-3p				miR-93-3p/105			
	Both low	One	Both high	*P*	Both low	One	Both high	*P*
Local metastasis				0.086				0.676
No	209	314	183		173	355	178	
Yes	143	284	166		157	295	141	
Distal metastasis				0.487				0.006*
No	260	425	259		256	476	212	
Yes	92	173	90		74	174	107	
Grade				0.002*				0.063
1	25	59	22		32	57	17	
2	163	221	118		127	264	111	
3	144	289	194		153	296	178	
Null	20	29	15		18	33	13	
Stage				0.186				0.344
0	3	4	6		4	3	6	
1	107	176	104		101	197	89	
2	151	299	168		163	293	162	
3	25	45	30		20	58	22	
4	4	4	2		2	6	2	
Null	62	70	39		40	93	38	

### miR-105/93-3p stimulated Wnt/β-catenin signaling through downregulation of SFRP1 and confer cisplatin resistance

**Fig. 3:**
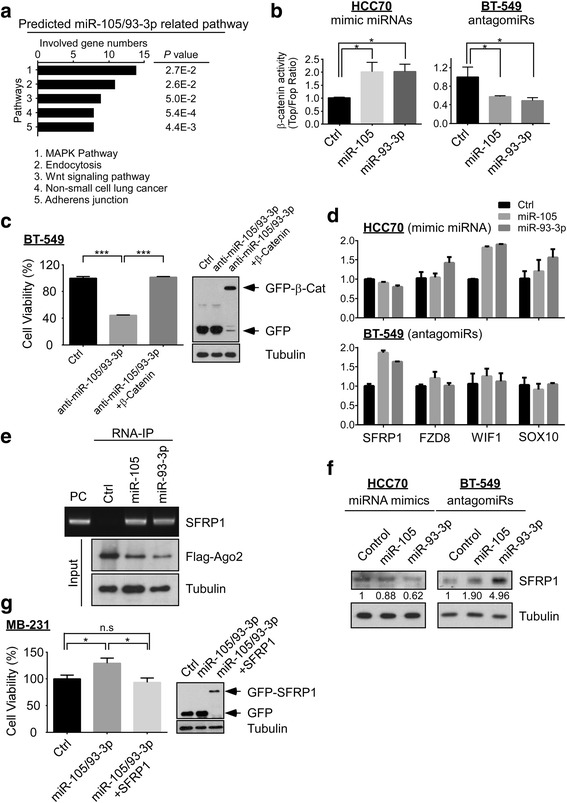
miR-105/93-3p activates Wnt/β-catenin signaling through downregulation of SFRP1 to promote cisplatin resistance. **a** Bioinformatic analysis was performed with the DAVID online tool to identify potential cell signaling pathways that may be modulated by miR-105/93-3p. **b** miR-105/miR-93-3p were overexpressed in HCC70 or silenced in BT-549 to examine β-catenin activity using the TOP/FOP reporter assay 48 hours post transfection. **c** Silencing of miR-105/93-3p with or without β-catenin overexpression in BT-549 cells to determine cell viability after 20 μM cisplatin treatment. Treatments were normalized to control. **d** Ectopic overexpression or knockdown of miR-105 or miR-93-3p to examine the potential target genes of miR-105 and miR-93-3p by qPCR. **e** Ectopic overexpression of Flag-Ago2 with indicated miRNAs in 293 T cells. Flag antibody was used to precipitate miRNA-Ago2-mRNA complex. The expression of SFRP1 mRNA in complex with miR-93-3p and miR-105 was determined by PCR. *PC* positive control. **f** Overexpression or knockdown of indicated miRNAs to determine SFRP1 expression level by Western blot at 48 hours post-transfection. **g** Ectopic overexpression of miR-105/93-3p with or without SFRP1 in MB-231 cells to examine cell viability after 20 μM cisplatin treatment. Treatments were normalized to control. Mean ± SEM; ^*^ *P* < 0.05, ^***^ *P* < 0.001, *n.s.* no significance

In order to investigate the underlying oncogenic mechanisms of miR-105/93-3p, a list of potential target genes for miR-105 and miR-93-3p was generated using RNA22, RNAhybrid and Targetscan. Additional file 1: Figure S4A shows that combined results from the three platforms identified 512 genes that can potentially be bound by both miR-105 and miR-93-3p on their 3′UTR. Further analyzing these target genes by DAVID, an online tool for functional annotation, MAPK and Wnt/β-catenin signaling pathways were predicted to be two of the potential targets of miR-105/93-3p-related signaling (Fig. 3a). In addition, IPA was conducted on the original mRNA profiling data from the same patient samples, this time segregated by miR-105 or miR-93-3p expression levels. This analysis revealed that Wnt/β-catenin signaling was also among the highest ranking potential pathways to be modulated in patient samples with high expression of either miRNA (Additional file 1: Figure S4B). Ectopic overexpression or silencing of miR-105/93-3p in TNBC cells were then used to examine both MAPK and Wnt/β-catenin signaling in response to miRNA modulation. MAPK signaling was probed by immunoblotting and Wnt/β-catenin by a TOP/FOP reporter assay. Figure 3b and Additional file 1: Figure S4C show that miR-93-3p overexpression can significantly induce β-catenin activity up to twofold (*P* = 0.013) whereas the miR-93-3p knockdown decreased β-catenin activity to approximately 50% of the control value (*P* = 0.017). Similarly, miR-105 activated β-catenin activity up to twofold (miR-105 overexpression) and decreased β-catenin activity to half of the control level (miR-105 knockdown). Interestingly, overexpression or silencing of miR-105 or miR-93-3p did not affect ERK phosphorylation or AKT phosphorylation, indicating that miR-105/93-3p does not affect MAPK signaling in TNBC cells (Additional file 1: Figure S4D). β-catenin was then overexpressed in miR-105/93-3p-silenced BT-549 cells to investigate the functional effects on cisplatin-mediated cell death. Figure 3c shows that miR-105/93-3p knockdown can increase cisplatin (10 μM)-mediated cell death by about 60% (*P* < 0.0001), however, this effect can be overcome by restoration of β-catenin (*P* < 0.0001). These results support the conclusion that miR-105/93-3p enhances chemoresistance in TNBC through β-catenin activation.

Two possibilities that might explain miR-105/93-3p-mediated β-catenin activation were the downregulation of upstream negative regulators and the upregulation of upstream positive regulators. First, nine genes related to Wnt/β-catenin signaling were predicted to be altered miR-105/93-3p from the RNA22, RNAhybrid and Targetscan analyses. These nine genes are listed in Additional file 1: Figure S5A. Second, the IPA analysis of patient data indicated five candidate genes, which were involved in Wnt/β-catenin signaling might be mediated by miR-105 (and five genes by miR-93-3p) shown in Additional file 1: Figure S5B. Interestingly, SFRP1, a Wnt/β-catenin signaling pathway suppressor, was identified from predicted miRNA target genes and miRNA-associated mRNA profiling from patients (Additional file 1: Figure S5A–B). Third, these potential target genes were examined after overexpressing or silencing miR-105 and miR-93-3p in breast cancer cell lines. The data showed that among all of the tested mRNA levels, only SFRP1 was decreased in miR-105 or miR-93-3p-overexpressing cells and increased in miR-105 or miR-93-3p silenced cells (Fig. 3d). Finally, both miR-105 and miR-93-3p showed strong binding potential on the 3′UTR of SFRP1 (binding energy was -27.2 and -28.5 kcal/mol, respectively), as predicted by RNAhybrid (Additional file 1: Figure S5C). Therefore, we hypothesized that miR-105/93-3p might directly target and downregulate SFRP1, resulting in activation of Wnt/β-catenin signaling.

Ago2-mediated RNA-IP and immunoblotting experiments were then used to confirm the interaction between miR-105 (or miR-93-3p) and SFRP1. In cells that co-overexpress either miR-105 or miR-93-3p with Ago2, SFRP1 mRNA was found to bind both miR-105 and miR-93-3p (Fig. 3e). Moreover, ectopic overexpression miR-105 or miR-93-3p decreased SFRP1 protein levels in HCC70 (0.88- and 0.63-fold, respectively) and 293 T (0.69- and 0.48-fold, respectively) cells. Furthermore, silencing miR-105 or miR-93-3p increased SFRP1 protein levels in BT-549 (1.90- and 4.96-fold, respectively) and MB-231 (7.21- and 19.7-fold, respectively) cells (Fig. 3f and Additional file 1: Figure S5D). These results support the conclusion that miR-105 and miR-93-3p bind to the 3′UTR of SFRP1 and consequently decrease SFRP1 protein levels, leading to activation of Wnt/β-catenin signaling in TNBC cells. Because SFRP1 was previously reported to inhibit chemoresistance [27], we further investigated the functional effects of SFRP1 in miR-105/93-3p-mediated chemoresistance by restoring SFRP1 in miR-105/93-3p-overexpressing MB-231 cells. Cisplatin-mediated cell death was attenuated in the miR-105/93-3p-overexpressing cells (*P* = 0.027) and co-overexpression of SFRP1 with miR-105/93-3p can almost completely reverse the miR-105/93-3p-mediated effect on cell death (miR-105/93-3p versus miR-105/93-3p + SFRP1, *P* = 0.013; Control versus miR-105/93-3p + SFRP1, *P* = 0.56) (Fig. 3g). In summary, miR-105 and miR-93-3p can directly suppress SFRP1 and lead to activation of Wnt/β-catenin signaling, conferring chemoresistance and stemness to TNBC cells.

### miR-105/93-3p was highly expressed in TNBC patients and predicted TNBC survival

**Fig. 4:**
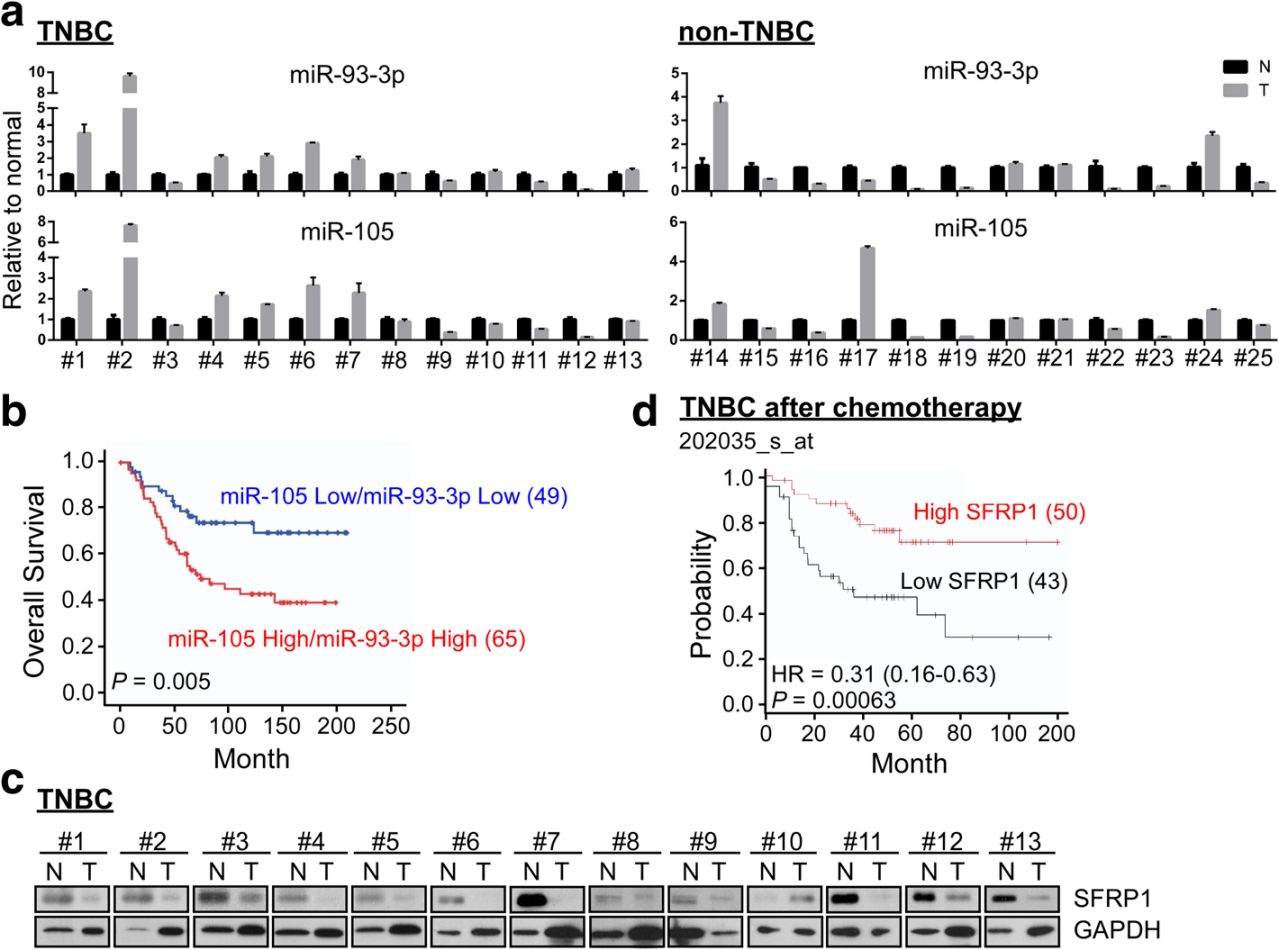
miR-93-3p/105 act as a predictive biomarker for TNBC. **a** miR-93-3p and miR-105 levels were determined in 13 TNBC and 12 non-TNBC N-T pairs by qPCR. **b** Overall survival of 114 triple negative breast cancer patients was analyzed by Kaplan-Meier analysis after stratification by miR-93-3p/miR-105 expression. **c** SFRP1 levels were determined in 13 TNBC N-T paired tissues by immunoblotting. **d** Overall survival of 93 triple negative breast cancer patients that had previously received chemotherapy was obtained from the Kaplan-Meier plotter website. Using the median level as a cutoff, samples were stratified by SFRP1 levels to test the correlation with overall survival. *TNBC* triple negative breast cancer

In order to test whether miR-105 and miR-93-3p were upregulated specifically in cancerous tissue of TNBC patients, we measured the miRNA levels in 13 TNBC normal and tumor tissue (N-T) pairs by qPCR and compared with that of non-TNBC patients (12 non-TNBC N-T pairs). Figure 4a showed higher levels of miR-93-3p (8 of 13 N-T pairs, 62%) were measured in tumor tissues than normal tissues, with miR-105 measurements producing similar results (6 of 13 N-T pairs, 46%). In contrast, only 4 of 12 patients had higher expression levels of miR-105 and miR-93-3p in non-TNBC tumor tissue compared to normal (Fig. 4a). To investigate the prognostic role of miR-105/93-3p in TNBC, Kaplan-Meier analyses were performed using the TNBC cohort from the Metabric database. As expected, the patients with high miR-105/93-3p levels showed the positive correlation with poor survival (*P* = 0.005) (Fig. 4b). Interestingly, our result also showed that SFRP1 protein levels were inversely correlated with miR-105/93-3p expression levels in tissue from TNBC N-T pairs, but not non-TNBC N-T pairs. The contingency plot showed that 100% of high miR-93-3p (or miR-105) levels correlated with low SFRP1 protein levels in TNBC, but only 2 of 4 high miR-93-3p (or miR-105) levels correlate with low SFRP1 protein levels in non-TNBC (Fig. 4c and Additional file 1: Figure S6A and S6B). Importantly, Kaplan-Meier analysis [28] of this cohort showed that low SFRP1 expression was associated with poor survival in TNBC patients, even after chemotherapy treatment (Fig. 4d and Additional file 1: Figure S6C). These results strongly suggest that SFRP1 can act as a prognostic predictor in TNBC. Collectively, these data support the overall hypothesis that miR-105/93-3p act to downregulate SFRP1 and thereby promote chemoresistance finally resulting in poor survival outcomes for TNBC patients.

### Circulating miR-105/93-3p serve as biomarkers to predict TNBC

**Fig. 5:**
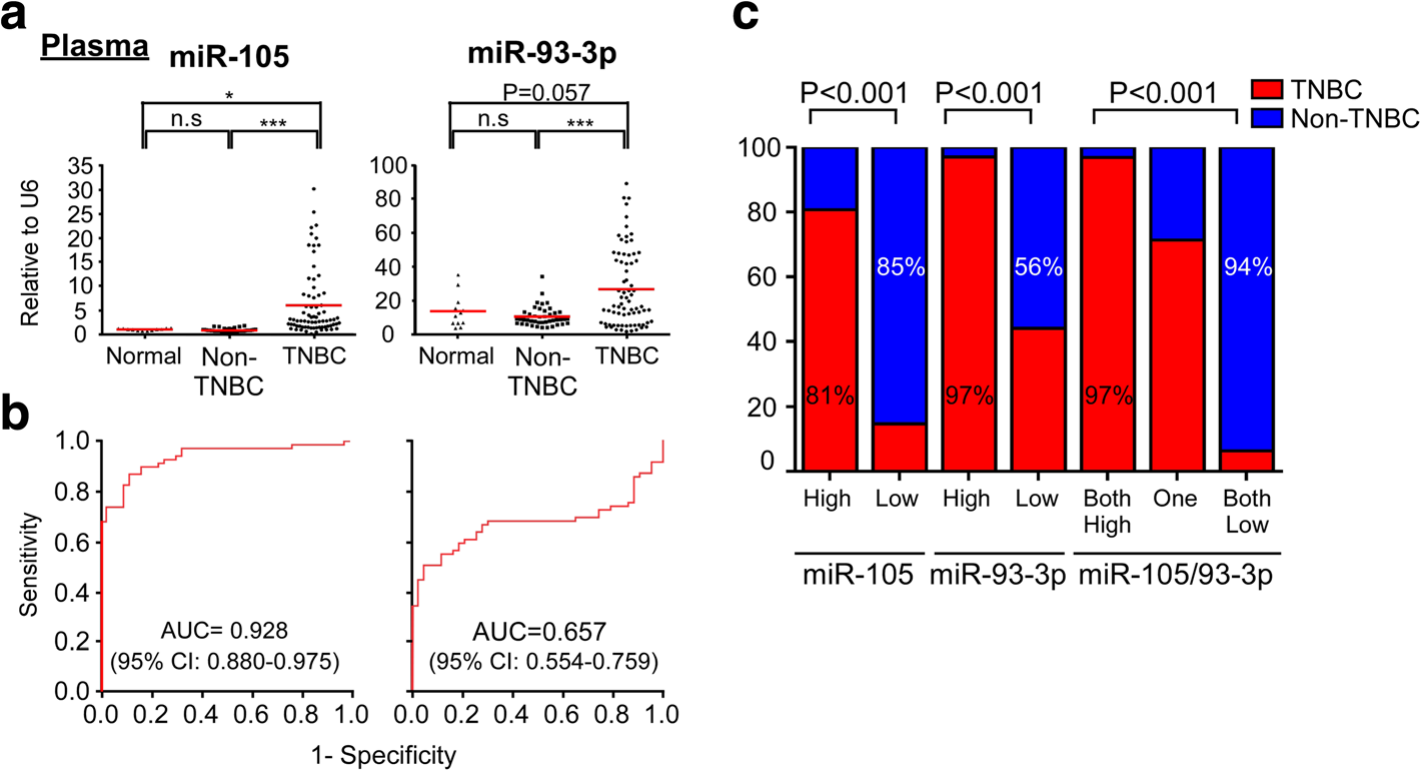
Circulating miR-105/miR-93-3p serves as diagnostic biomarker for TNBC patients. **a** Circulating miR-105 and miR-93-3p were measured in 12 normal and 118 breast cancer patient plasma samples (74 TNBC and 44 non-TNBC patients) by qPCR. **b** ROC curves were plotted to evaluate the predictive power of circulating miR-93-3p and miR-105 for the TNBC subtype. **c** A contingency plot showing the distribution of TNBC and non-TNBC patients with the indicated circulating miRNA levels. The correlation was examined by chi-square test. Mean ± SEM; ^*^ *P* < 0.05, ^***^ *P* < 0.001, *n.s.* no significance. *TNBC* triple negative breast cancer

Accumulating studies emphasize the diagnostic and prognostic importance of circulating miRNAs [16–18]. To explore the possibility that circulating miR-105 and miR-93-3p may act as a diagnostic biomarker for TNBC, circulating miR-105 and miR-93-3p levels were examined in plasma from 12 healthy controls and 118 breast cancer (74 TNBC and 44 non-TNBC) patients. The expression level of miR-105 and miR-93-3p were normalized to U6 in plasma. miR-105 and miR-93-3p levels were significantly elevated in the plasma of TNBC patients compared with non-TNBC patients (*P* < 0.001 and *P* < 0.001, respectively) or healthy controls (*P* = 0.014 and *P* = 0.057, respectively) (Fig. 5a). Notably, the plasma levels of miR-105 and miR-93-3p showed no difference between non-TNBC and normal group, indicating that circulating miR-105 and miR-93-3p may specifically act as a diagnostic marker for TNBC. In order to evaluate the predictive power of circulating miR-105 and miR-93-3p, a ROC curve was generated, and the data showed that circulating miR-105 and miR-93-3p have the strong predictive ability for the TNBC subtype. The corresponding AUC was 0.928 (95% CI 0.880–0.975) and 0.657 (95% CI 0.554–0.759), for circulating miR-105 and miR-93-3p respectively, indicating that either miRNA could serve as a good diagnostic biomarker for TNBC (Fig. 5b). miR-93-3p and miR-105 using the cutoff values identified by ROC curves (cut point of miR-105 = 1.31 and miR-93-3p = 19.23) to evaluate the specificity and accuracy. The contingency plot indicated that high circulating miR-105 (81%) and miR-93-3p (97%) levels were significantly associated with TNBC subtype, and high expression of circulating miR-105/93-3p (97%) also showed a high correlation with TNBC subtype (Fig. 5c). Notably, a combination of miR-105/93-3p increase the specificity to 94% from 85% (miR-105) and 56% (miR-93-3p). Collectively, circulating miR-105/93-3p may provide a powerful diagnostic biomarker for TNBC.

## Discussion

TNBC is often diagnosed in an advanced stage and accompany with poor survival. Developing diagnostic approaches may help to benefit clinical outcome in patients. However, it is still a lack of diagnosis marker for TNBC patients with early disease. In the current study, elevated levels of miR-105 and miR-93-3p were detected in both TNBC primary tissue and plasma, but not in tissues from non-TNBC patients or healthy controls (Figs. 4 and 5). Importantly, high level of circulating miR-105 and miR-93-3p were showed the strong predictive ability for the TNBC subtype, providing the opportunity of liquid biopsy for TNBC patients. Further analyzing the functional roles of miR-105 and miR-93-3p, we showed that miR-105 and miR-93-3p can promote chemoresistance in TNBC cells (Fig. 2). Moreover, we demonstrated that miR-105 and miR-93-3p can activate Wnt/β-catenin signaling by decreasing SFRP1, and thereby confer chemoresistance to TNBC cells (Fig. 3). Together, the data led us to conclude that miR-105 and miR-93-3p both in tissue and plasma can sever as a diagnostic and predictive marker for TNBC patients.

Numerous miRNAs have been reported to modulate progression, metastasis and chemoresistance in breast cancer [29]. For example, miR-18a downregulates PTEN and HOXA1 to promote breast cancer progression [30]. miR-22 indirectly activates ZEB1/2 to promote metastasis [12]. miR-374a activates Wnt/β-catenin signaling to promote breast cancer metastasis [8], and miR-125b promotes breast cancer chemoresistance by maintaining cancer stem-like cells [31]. In addition, some of miRNAs were reported to serve the role of either tumor suppressor or oncogene in TNBC such as miR-200 family, miR-211-5p, and miR-143-3p serve as tumor-suppressive miRNAs [32–34]. In this study, we demonstrated that miR-93-3p and miR-105 increase β-catenin activity by downregulating SFRP1 in TNBC (Fig. 3). Notably, the downregulation of SFRP1 is most sensitive to miR-93-3p upregulation, whereas miR-105 showed comparatively limited effects. However, the downstream effects on β-catenin activity are very similar in cells with miR-105 or miR-93-3p overexpression or silencing. These results suggest that miR-105 may operate through multiple mechanisms to activate Wnt/β-catenin signaling in TNBC. miR-105 was reported to serve as tumor-suppressive miRNAs in glioma by targeting SOX9 and SUZ12, as well as in hepatocellular carcinoma by targeting NCOA1 [35–37]. However, secreted miR-105 has been previously found to disrupt the vascular endothelial cell barrier and as such, has been linked to breast cancer metastasis [38]. These observations suggest that miR-105 may possess other functional mechanisms in breast cancer, which remain to be investigated in the future.

Previously, Dey and colleagues reported that the Wnt/β-catenin pathway is activated in TNBC subtypes and provided evidence that increased Wnt/β-catenin signaling is associated with high grade, poor prognosis, and metastatic disease in several types of human cancers including breast cancer [39]. Activation of Wnt/β-catenin signaling promotes proliferation, stemness, and metastasis in breast cancer, and blocking Wnt/β-catenin can repress stemness and metastasis in over-activated cells [8, 40–42]. Our results showing that miR-105/93-3p acts through the SFRP1/Wnt/β-catenin axis to increase stemness, chemoresistance and metastasis in TNBC are in agreement with these previous studies (Figs. 2 and 3).

Our data suggest that elevated miR-105 and miR-93-3p in both tumor tissue and plasma can act as a predictive biomarker for TNBC (Figs. 4 and 5 and Table 2). Circulating miRNAs have several advantages for using as biomarkers, including non-invasive sampling, high stability and ease of detection [18, 43]. Recently, the diagnostic, predictive and prognostic capability of circulating miRNAs in breast and lung cancer has been explored. For example, a three-miRNA combination (miR-199a, miR-29c, and miR-424) may act as an early detection biomarker for breast cancer. Furthermore, it has been proposed that miR-199a-3p can serve as TNBC diagnostic biomarker [44–46]. Herein, we identify circulating miR-105/93-3p as specifically increased in TNBC patients, but not in non-TNBC patients or healthy controls, suggesting that circulating miR-105/93-3p may serve as a powerful diagnostic biomarker for TNBC (Fig. 5). Our cohort contained 46 patients in early stage (stage I/II) disease and 21 patients in advanced disease (stage III/IV) but lost clinical information in other 51 patients. Our data also suggest that circulating miR-105/93-3p also can be used to identify early-stage TNBC. The corresponding AUC was 0.939 in stage I/II TNBC patients (Additional file 1: Figure S6D). TNBC is often diagnosed with high grade and the 5-year survival rate is therefore greatly decreased. Early-stage diagnosis has a much better survival rate (76–93%) when compared to late-stage diagnosis (15–45%) of TNBC [2, 7]. Our data show that combined circulating miR-105/93-3p level is a promising candidate to serve as a non-invasive and early-stage detection biomarker for TNBC. Discovery and development of a reliable early detection biomarker is a critical need for TNBC patients and would almost certainly improve clinical outcome.

Circulating miRNAs are not only useful as biomarkers but also may be viable therapeutic targets since they represent a mechanism of communication between cells that can promote cancer progression or metastasis. For example, metastatic breast cancer cells that secrete exosome-embedded miR-105 act to destroy tight junctions, ZO-1, and trigger vascular endothelial cells to progress toward a pro-metastatic niche [38]. Exosomes containing several components, including miRNA, lncRNA and proteins, can modulate metastasis or drug response in cancer [47, 48]. Together with our findings, suggesting that circulating miR-105 and miR-93-3p may be taken up by surrounding tumor cells wherein they may activate Wnt/β-catenin signaling and lead to chemoresistance in TNBC patients. Collectively, our evidence highlights the importance of miRNA in tumor progression and diagnostic, predictive, and prognostic roles. Currently, several miRNAs, such as MRX34, antagomiR-221 and antagomiR-10b, represent a new class of therapeutic targets for cancers [49]. The present study suggests that miR-105/93-3p may not only act as a biomarker for TNBC but also can be considered as targets for developing new therapies in TNBC.

## Conclusions

The purpose of this study is to identify miRNAs that are specifically associated with poor survival in TNBC and determine the underlying mechanism by which they contribute to carcinogenesis. Overall, we uncovered a novel mechanism of miR105/93-3p-mediated chemoresistance and revealed that circulating miR-105/93-3p serve as powerful diagnostic biomarkers for TNBC. Circulating miRNAs are promising biomarkers in cancer because of highly stability and non-invasive biopsy. Useful biomarkers provide a means for diagnosis or personalized treatment.

Additionally, miRNAs have functional relevance in cancer, and as such, may have further utility as treatment targets. Current therapies for TNBC only generate 20–30% pathological complete response, and there are no early detection biomarkers for this disease. In this study, we found that miR-105 and miR-93-3p were specifically upregulated and correlated with poor survival by increasing stemness, chemoresistance, and metastasis in TNBC. Moreover, we identified that miR-105/93-3p activate Wnt/β-catenin signaling through SFRP1 downregulation leading to chemoresistance in TNBC.

Our study provides the first evidence that circulating miR-93-3p/105 serve as a diagnostic biomarker for TNBC and elucidates the underlying mechanisms by which these miRNAs contribute to carcinogenesis. These results are important for early detection and personalized treatment of TNBC patients.

## Additional files

Additional file 1: Figure S1. Association between identified miRNAs and overall survival in TNBC and non-TNBC patients. **A** Identification of dysregulated miRNAs in TNBC compared with non-TNBC using Student *t* test. **B** Elevated expression of miR-301b, miR-181a-3p, miR-105, and miR-93 were individually associated with poor overall survival in 1095 non-triple negative breast cancer patients by Kaplan-Meier analysis. The correlation between indicated miRNAs and overall survival in **(C)** 204 TNBC patients and **(D)** 1095 non-TNBC patients was analyzed by Kaplan-Meier analysis. **E** Indicated miRNAs expression levels were examined in the independent cohort (GSE40267, N = 173), which contained 94 TNBC, 79 non-TNBC. **Figure S2** miR-105 and miR-93-3p promote cellular migration but not proliferation. The effect of ectopic overexpression or silencing of indicated miRNAs on cell proliferation as determined by **(A)** colony-forming assay and **(B)** MTT assay. **C** The effect of ectopic overexpression or silencing of indicated miRNAs on cell migration ability as measured by the Boyden chamber transwell migration assay. **D** The miRNA-overexpressing HCC70 cells and miRNA silenced-BT-549 cells were seeded into matrigel-coated transwells to evaluate cell invasion in vitro. **Figure S3** miR-105 and miR-93-3p confer cisplatin resistance. Cisplatin was administered with the indicated dose to cells with **(A)** ectopic overexpression or **(B)** silencing of indicated miRNAs prior to determining cell viability by the MTT assay. **C** Co-transfection with miR-105 and miR-93-3p antagomiRs in HCC1937 followed by measurement of the cisplatin response by MTT assay. **D** miR-105/93-3p co-silenced-BT-549 cells were seeded at 10,000 cells/ml into an ultra-low attachment plate for 10 days to evaluate mammosphere formation, as an indicator of stemness. Relative efficiency of mammosphere formation was measured in control and miR-105/93-3p-knockdown BT-549 cells. **Figure S4** miR-105 and miR-93-3p activate Wnt/β-catenin signaling. **A** Bioinformatic analysis to identify potential miR-105 and miR-93-3p target genes. **B** The miR-105 and miR-93-3p associated mRNA profiling, paired with miRNA microarrays from the same patients were analyzed by IPA. **C** Ectopic overexpression of miR-105 or miR-93-3p and subsequent determination of β-catenin activity by the TOP/FOP reporter assay. **D** Immunoblotting was performed to measure levels of the indicated proteins in miR-105 or miR-93-3p manipulated TNBC cells. **Figure S5** miR-93-3p/105 target to SFRP1 and decrease SFRP1 expression level. **A** Bioinformatic prediction of target genes for miR-105/93-3p involved in Wnt/b-catenin signaling. **B** IPA was performed to identify potential upstream regulators of Wnt/β-catenin that are modulated in TNBC with high expression of miR-105 or miR-93-3p. **C** RNA hybrid was performed to examine the binding energy between miR-105 or miR-93-3p with 3′-UTR of SFRP1. **D** Ectopic overexpression or silencing of indicated miRNAs followed by determination of SFRP1 protein levels by immunoblotting. **Figure S6** miR-93-3p/105 can serve as biomarker for early TNBC patients and inversely correlated with SFRP1 in TNBC but not in non-TNBC patients. **A** The SFRP1 levels were determined in 12 non-TNBC N-T paired tissues by immunoblotting. **B** The contingency plot showed the distribution of SFRP1 in the indicated circulating miRNA levels. **C** Overall survival of 249 TNBC patients and 93 TNBC patients with chemotherapy, those were obtained from Kaplan-Meier plotter website were stratified with SFRP1 by Kaplan-Meier analysis. **D** Combination of circulating miR-93-3p/105 to evaluate the predictive power for stage I/II (N = 46) or stage III/IV (*N* = 21) stage of TNBC by ROC curve. (PDF 29884 kb)

Additional file 2: Table S1. Dysregulated miRNAs in TNBC patients. (DOCX 13 kb)

Additional file 3: Table S2. Univariate and multivariate analysis of clinical features and four oncomiRs associated with overall survival. (DOCX 12 kb)

## Abbreviations

Ago2: Agonature 2
AUC: Area under the curve
ER: Estrogen receptor
HER2: Human epidermal growth factor receptor 2
HR: Hazard ratio
miRNA: MicroRNA
pCR: Pathologic complete response
PR: Progesterone receptor
ROC curve: Receiver operating characteristic curve
SFRP1: Secreted frizzled related protein 1
TNBC: Triple negative breast cancer
