# Supplementary material for: miR-105/93-3p promotes chemoresistance and circulating miR-105/93-3p acts as a diagnostic biomarker for triple negative breast cancer
Source: Breast Cancer Res. 2017 Dec 19;19:133. doi: 10.1186/s13058-017-0918-2 (PMC5738224; doi:10.1186/s13058-017-0918-2)

Supplementary Figure 1

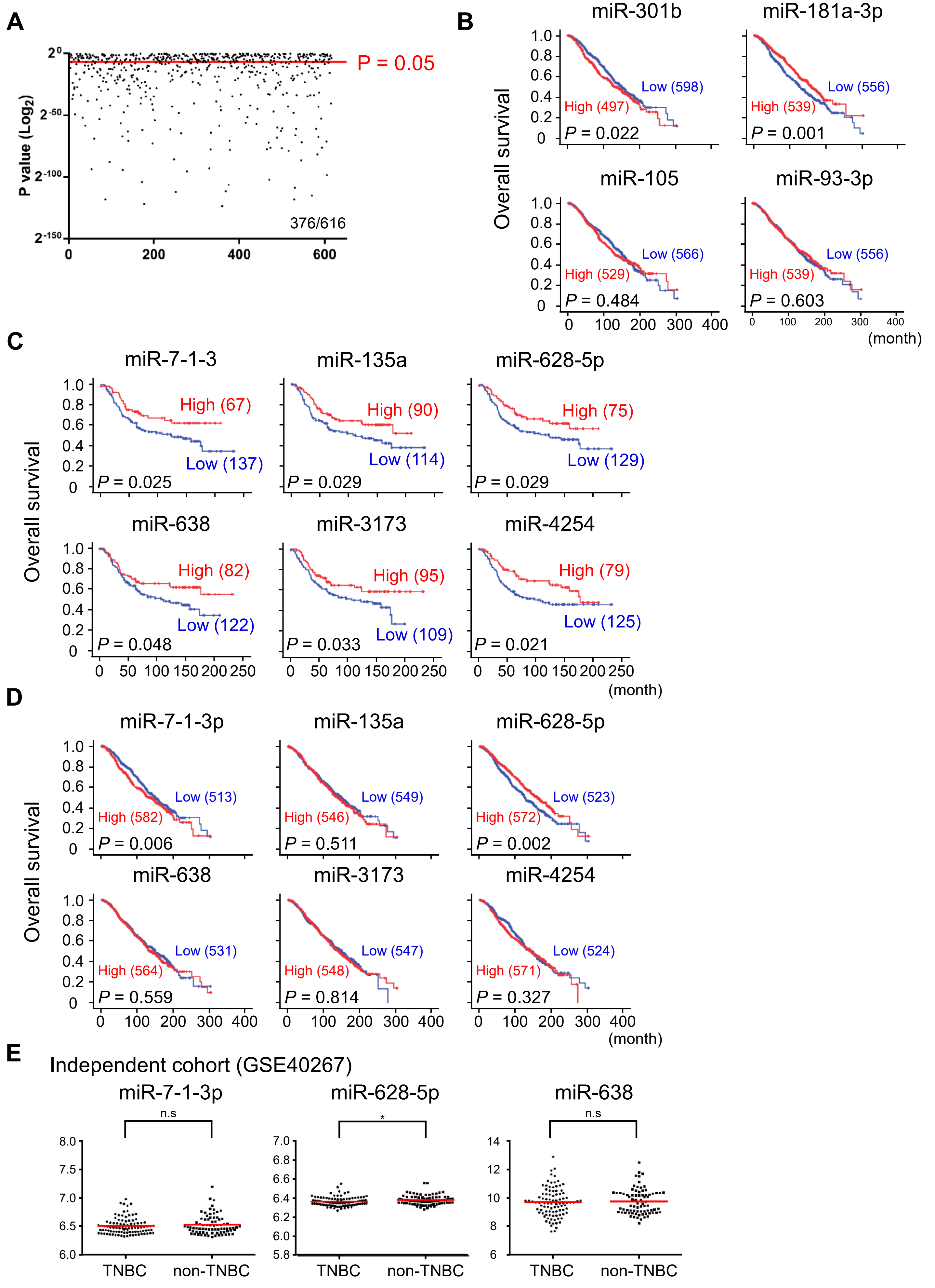

Supplementary Figure 2

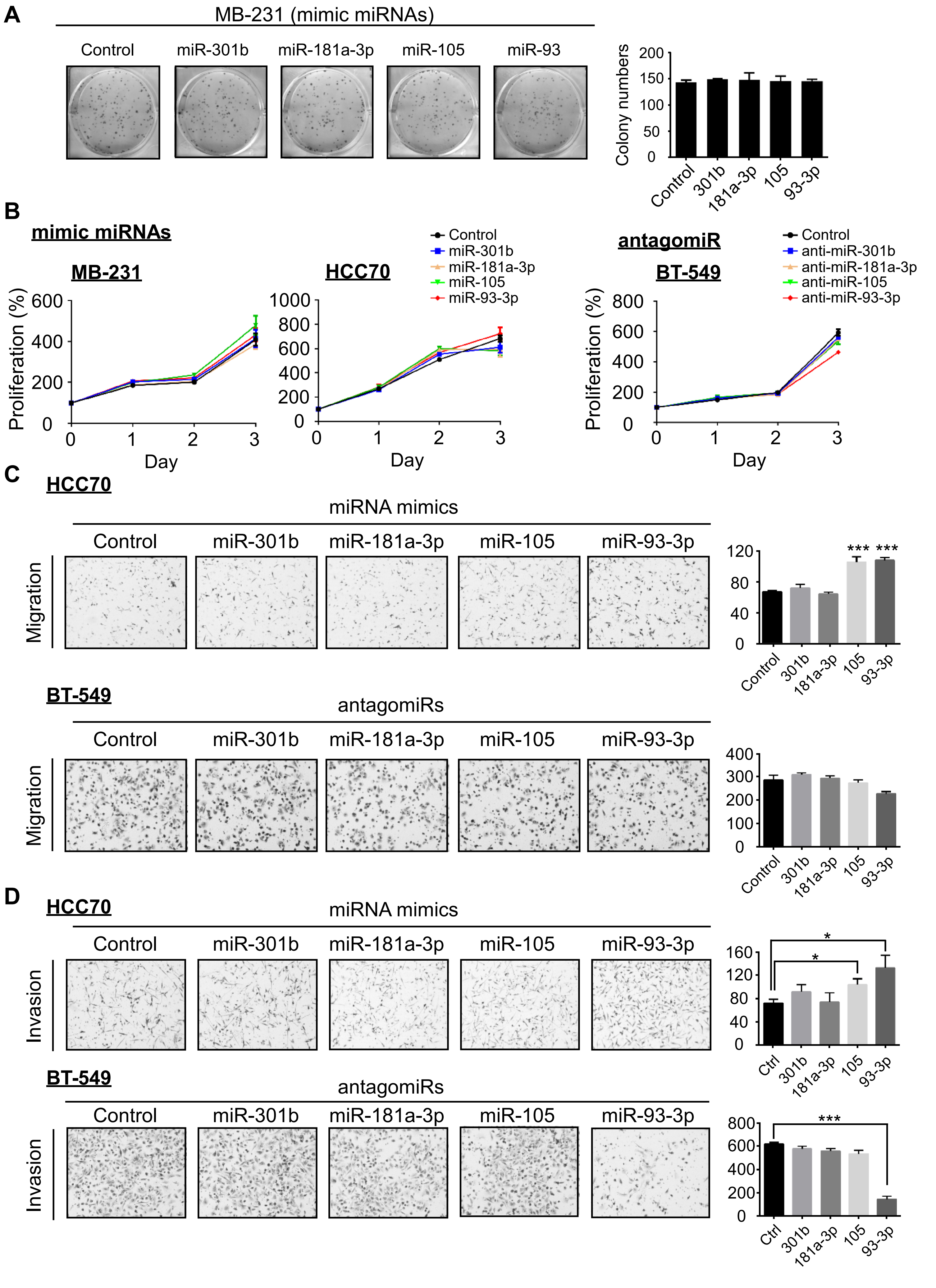

Supplementary Figure 3

A

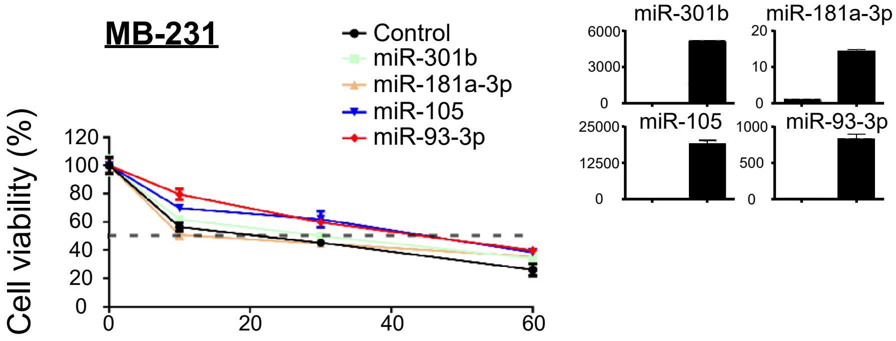

B

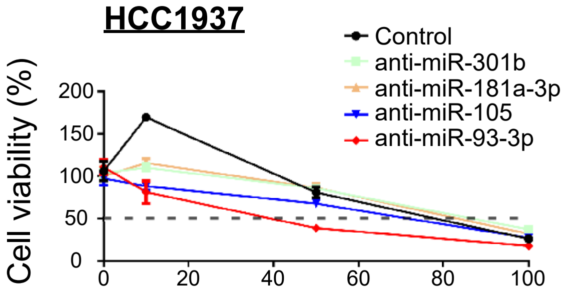

C

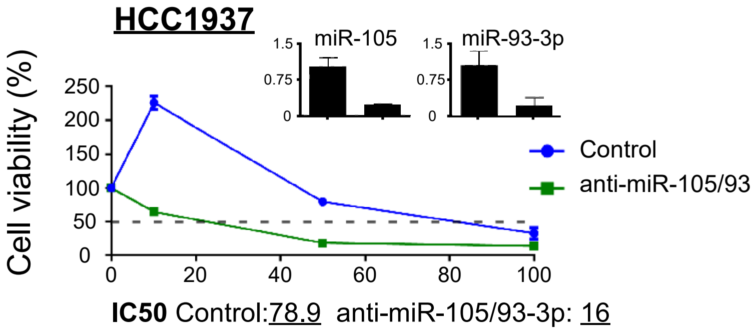

D

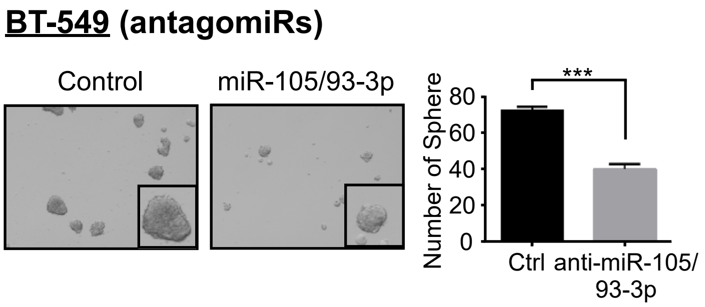

Supplementary Figure 4

A

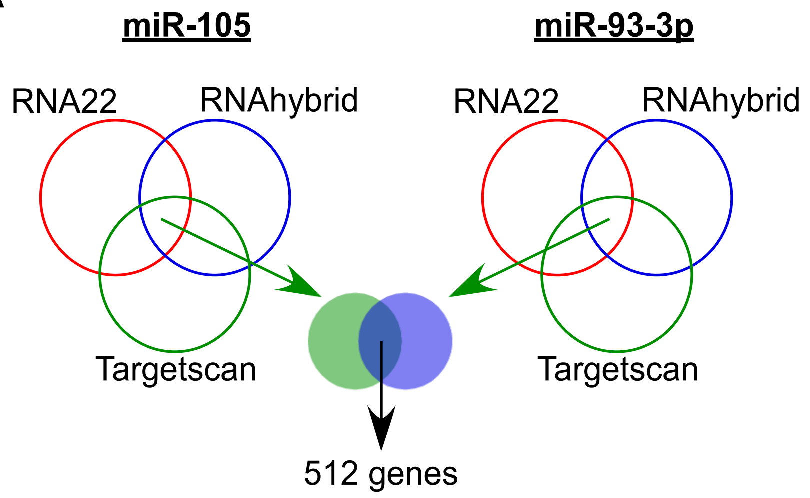

C

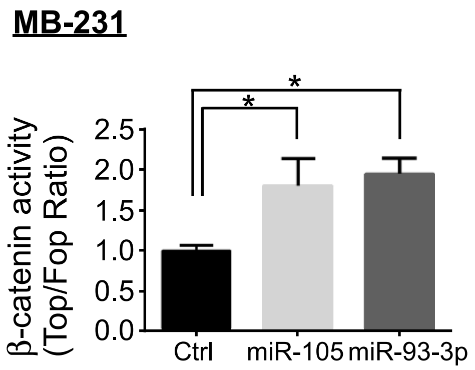

B

miR-105

Top Canonical Pathways

Name	p-value	Overlap
Bladder Cancer Signaling	4.83E-04	4.6 % 4/87
Colorectal Cancer Metastasis Signaling	5.19E-04	2.5 % 6/241
Wnt/-catenin Signaling	7.32E-04	2.9 % 5/170
Xenobiotic Metabolism Signaling	1.01E-03	2.2 % 6/274
Thyroid Hormone Metabolism II (via Conjugation and/or Degradation)	1.66E-03	5.4 % 3/56

miR-93-3p

Top Canonical Pathways

Name	p-value	Overlap
Wnt/-catenin Signaling	6.96E-04	2.9 % 5/170
Cell Cycle Control of Chromosomal Replication	5.52E-03	7.4 % 2/27
Agranulocyte Adhesion and Diapedesis	7.94E-03	2.1 % 4/190
Leukocyte Extravasation Signaling	1.01E-02	2.0 % 4/204
Protein Ubiquitination Pathway	2.24E-02	1.5 % 4/259

D

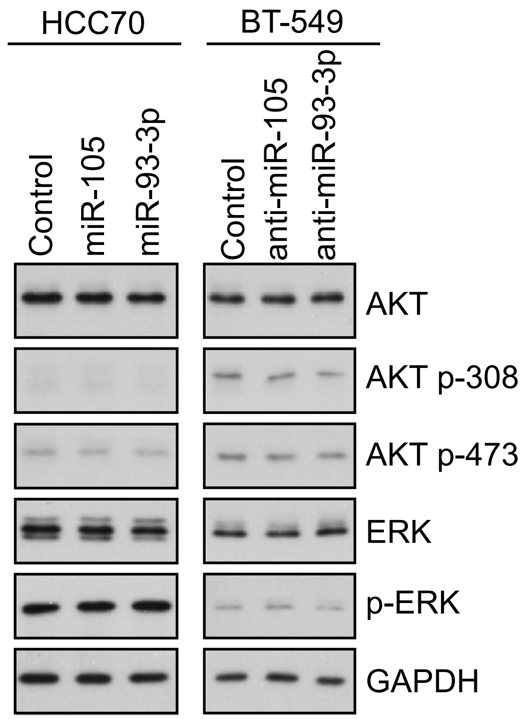

Supplementary Figure 5

A

Predicted miRNA target genes in Wnt signaling pathway	
FZD3	**SFRP1**
LRP6	SMAD2
NFATC3	SMAD3
PRKCA	
PRKX	
PRICKLE2	

B

miR-105		miR-93-3p	
Symbol	Expected	Symbol	Expected
CDH1		CDH3	
CDH3		MMP7	Up
FZD8	Up	**SFRP1**	Down
**SFRP1**	Down	SOX10	Down
WIF1	Down	UBD	

C

miR-105 **free energy= -27.2 kcal/mol**

Position 974

```
SFRP1 5' C           UUAUUUUUCA     UUUUAAAAC     UACAU            A 3'
          CCAC                 AGG           AGUC       UGAGCAUUUGA
          GGUG                 UCC           UCAG       ACUCGUAAACU
miRNA 3' U                                                            5'
```

miR-93-3p **free energy= -28.5 kcal/mol**

Position 323

```
SFRP1    5' G       UUGGGCGGAA       AG               C  3'
             GGGA              AGUG     AGC  CAGCAG
             CCCU              UCAC     UCG  GUCGUC
miRNA    3' G                       GA     A          A  5'
```

D

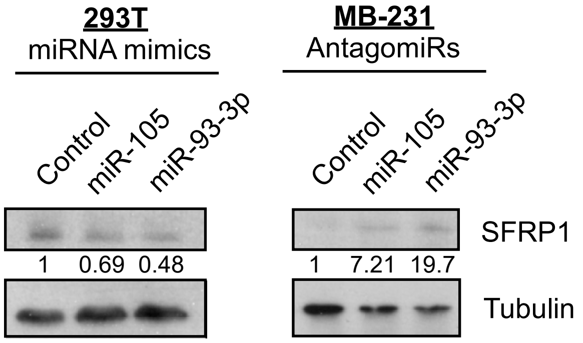

Supplementary Figure 6

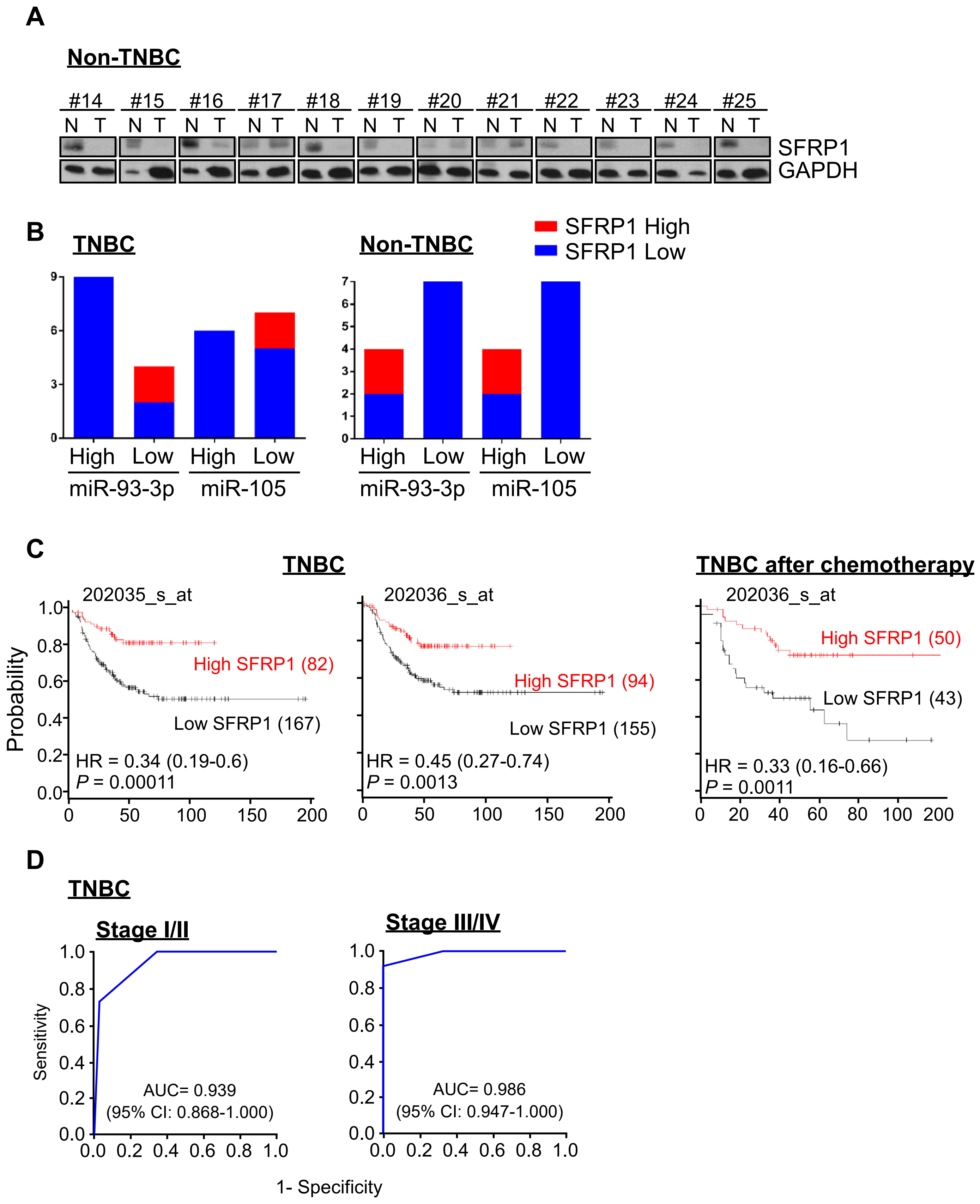

Supplement: Supplementary file 1 — Association between identified miRNAs and overall survival in TNBC and non-TNBC patients. A Identification of dysregulated miRNAs in TNBC compared with non-TNBC using Student t test. B Elevated expression of miR-301b, miR-181a-3p, miR-105, and miR-93 were individually associated with poor overall survival in 1095 non-triple negative breast cancer patients by Kaplan-Meier analysis. The correlation between indicated miRNAs and overall survival in (C) 204 TNBC patients and (D) 1095 non-TNBC patients was analyzed by Kaplan-Meier analysis. E Indicated miRNAs expression levels were examined in the independent cohort (GSE40267, N = 173), which contained 94 TNBC, 79 non-TNBC. Figure S2 miR-105 and miR-93-3p promote cellular migration but not proliferation. The effect of ectopic overexpression or silencing of indicated miRNAs on cell proliferation as determined by (A) colony-forming assay and (B) MTT assay. C The effect of ectopic overexpression or silencing of indicated miRNAs on cell migration ability as measured by the Boyden chamber transwell migration assay. D The miRNA-overexpressing HCC70 cells and miRNA silenced-BT-549 cells were seeded into matrigel-coated transwells to evaluate cell invasion in vitro. Figure S3 miR-105 and miR-93-3p confer cisplatin resistance. Cisplatin was administered with the indicated dose to cells with (A) ectopic overexpression or (B) silencing of indicated miRNAs prior to determining cell viability by the MTT assay. C Co-transfection with miR-105 and miR-93-3p antagomiRs in HCC1937 followed by measurement of the cisplatin response by MTT assay. D miR-105/93-3p co-silenced-BT-549 cells were seeded at 10,000 cells/ml into an ultra-low attachment plate for 10 days to evaluate mammosphere formation, as an indicator of stemness. Relative efficiency of mammosphere formation was measured in control and miR-105/93-3p-knockdown BT-549 cells. Figure S4 miR-105 and miR-93-3p activate Wnt/β-catenin signaling. A Bioinformatic analysis to identify potent [file 13058_2017_918_MOESM1_ESM.pdf]
