# Supplementary material for: miR-105/93-3p promotes chemoresistance and circulating miR-105/93-3p acts as a diagnostic biomarker for triple negative breast cancer
Source: Breast Cancer Res. 2017 Dec 19;19:133. doi: 10.1186/s13058-017-0918-2 (PMC5738224; doi:10.1186/s13058-017-0918-2)
Supplement: Supplementary file 2 — Dysregulated miRNAs in TNBC patients. (DOCX 13 kb) [file 13058_2017_918_MOESM2_ESM.docx]

**Table S1.** Dysregulated miRNAs in TNBC patients

|  | Log Rank p-value | Survival contribution  for TNBC patients |
| --- | --- | --- |
| **Up-regulated miRNAs** |  |  |
| hsa-miR-150 | 0.003 | Good |
| hsa-miR-584 | 0.009 | Good |
| hsa-miR-142-5p | 0.012 | Good |
| hsa-miR-142-3p | 0.03 | Good |
| hsa-miR-571 | 0.032 | Good |
| hsa-miR-93-3p | 0.04 | Poor |
| hsa-miR-181a-2-3p | 0.042 | Poor |
| hsa-miR-105-5p | 0.045 | Poor |
| hsa-miR-301b | 0.047 | Poor |
| hsa-miR-452 | 0.047 | Good |
|  |  |  |
| **Down-regulated miRNAs** | |  |
| hsa-miR-200a-3p | 0.008 | Poor |
| hsa-miR-196a-3p | 0.012 | Poor |
| hsa-miR-33b | 0.014 | Poor |
| hsa-miR-99b | 0.02 | Poor |
| hsa-miR-182 | 0.02 | Poor |
| hsa-miR-200b-3p | 0.02 | Poor |
| hsa-miR-182-3p | 0.021 | Poor |
| hsa-miR-663b | 0.021 | Poor |
| hsa-miR-135a | 0.022 | Good |
| hsa-miR-4254 | 0.023 | Good |
| hsa-miR-638 | 0.028 | Good |
| hsa-miR-628-5p | 0.029 | Good |
| hsa-miR-7-1-3p | 0.025 | Good |
| hsa-miR-181d | 0.042 | Poor |
| hsa-miR-3173 | 0.043 | Good |
| hsa-miR-141-3p | 0.043 | Poor |
