# Supplementary material for: miR-105/93-3p promotes chemoresistance and circulating miR-105/93-3p acts as a diagnostic biomarker for triple negative breast cancer
Source: Breast Cancer Res. 2017 Dec 19;19:133. doi: 10.1186/s13058-017-0918-2 (PMC5738224; doi:10.1186/s13058-017-0918-2)
Supplement: Supplementary file 3 — Univariate and multivariate analysis of clinical features and four oncomiRs associated with overall survival. (DOCX 12 kb) [file 13058_2017_918_MOESM3_ESM.docx]

**Table S2.** Univariate and multivariate analysis of clinical features and four oncomiRs associated with overall survival

|  | **Univariate** | | **Multivariate** | |
| --- | --- | --- | --- | --- |
| **Variables** | **HR (95% CI)** | **P** | **HR (95% CI)** | **P** |
| Lymph node metastasis | 1.80 (1.18-2.75) | **0.007** | 1.42 (0.92-2.19) | 0.117 |
| Distal metastasis | 7.12 (4.61-11.02) | **<0.001** | 7.98 (4.97-12.82) | **<0.001** |
| miR-301b | 1.19 (0.89-1.58) | 0.247 | 1.44 (1.02-2.04) | **0.041** |
| miR-181a-2-3p | 1.58 (0.99-1.53) | 0.055 | 1.02 (0.58-1.78) | 0.957 |
| miR-105-5p | 1.38 (0.90-2.13) | 0.14 | 1.60 (1.02-2.50) | **0.04** |
| miR-93-3p | 5.17 (1.11-24.08) | **0.036** | 2.16 (0.35-13.33) | 0.408 |
